# Spasmolytic Effect of *Stachys Lavandulifolia *Vahl. Crude Methanolic Extract and Fractions on Rat Ileum

**Published:** 2011

**Authors:** Mohammad Kazem Gharib Naseri, Neda Adibpour, Foroogh Namjooyan, Saeed Rezaee, Zahra Shahbazi

**Affiliations:** a *Physiology Research Center, Department of Physiology, Ahwaz Jundishapur University of Medical Sciences, Ahwaz, Iran.*; b * Department of medicinal Chemistry, School of Pharmacy, Ahwaz Jundishapur University of Medical Sciences, Ahwaz, Iran.*; c * Department of Pharmacognosy, School of Pharmacy, Ahwaz Jundishapur University of Medical Sciences, Ahwaz, Iran.*; d * Department of Pharmaceutics, School of Pharmacy, Ahwaz Jundishapur University of Medical Sciences, Ahwaz, Iran.*

**Keywords:** *Stachys lavandulifolia *Vahl., Rat, Ileum, Antispasmodic, Extract

## Abstract

The aim of this study was to investigate the effect of aerial parts of *Stachys lavandulifolia *Vahl. Extract on the rat ileum contractions. The crude extract was prepared by maceration method (90% methanol) followed by fractionating into chloroform, ethyl acetate, petroleum ether and water. In adult male Wistar rats, ileum was sectioned and mounted in tissue bath and their contractility was recorded is otonically. KCl (60 mM)- induced ileum contractions were attenuated by crude extract and its fractions. The most potent fraction was chloroformic fraction (CF) with IC_50_ 0.018 ± 0.126 = mg/mL. In calcium-free Tyrode solution with high K^+,^ the CF (0.01 – 0.04 mg/ml) attenuated CaCl_2_-induced contractions (p< 0.001). The CF (0.05-0.8 mg/mL) attenuated the carbachol-induced contraction (p<0.001). The CF antispasmodic effect was reduced by naloxone (as a non-selective opioid antagonist), not by propranolol and L-NAME as *β*-adrenoceptors antagonist and nitric oxide synthase inhibitor respectively. It was concluded that *S..lavandulifolia *can inhibit ileum contractility mainly via disturbing the calcium mobilization and partly by opioid receptors’ activation. Our results may support the traditional usage of this herb for treating diarrhea.

## Introduction

Diarrhea continues to be one of the leading causes of mortality and morbidity especially in children in developing countries (1). Diarrhea is characterized by hypermotility of gastrointestinal tract (2). Natural products have served as a medicinal source for centuries, and about half of the pharmaceuticals in use today are derived from natural products (3). *Stachys lavandulifolia* Vahl. from Lamiaceae is used in Iranian traditional medicine as an anxiolytic and to treat diarrhea (4). Furthermore, experimental studies have demonstrated the anxiolytic effect of* S. lavandulifolia* in mice (5, 6). Despite the extraction of *S. lavandulifolia* constituents such as *α*-pinene, myrcene, *β*-phellandrene and *β*-caryophyllene (4), its pharmacological effects on isolated smooth muscle have not been evaluated scientifically yet. Therefore, the aim of the present study was to investigate the antispasmodic potential of the *S. lavandulifolia *crude extract and its fractions on rat ileum.

## Experimental

*Plant material*

*Stachys lavandulifolia *Vahl. was collected (March 2008) from Zanjan province (center of Iran) and authenticated by Dr. Namjoyan from the Pharmacognosy Department of the Ahwaz Jundishapur University of Medical Sciences (AJUMS). A voucher specimen was deposited in the herbarium of the School of Pharmacy of AJUMS.

*Extraction and fractionation*

The aerial parts of the herb were dried in shade. The powder of the herb was macerated with methanol (90%) for 72 h at room temperature to obtain the crude extract (CE). The mixture was filtered (Whatman No.1) and the filtrate was concentrated by rotary evaporator and was air-dried in the next step (yield: 17.22%). Petroleum ether fraction (PF) was prepared from the CE (16.8 g) being mixed with 70 mL distilled water and 70 mL petroleum ether. The extraction was reported for three times followed by concentrating and air-drying (yield: 14.27%). In order to obtain the chloroformic fraction (CF), the PF was mixed with chloroform (70 mL) and then the mixture was concentrated and air-dried (yield: 1.74%). The same procedure was repeated in order to obtain ethyl acetate fraction (EF) using ethyl acetate (70 mL). The extraction process was reported for three times and the mixture was concentrated and thereafter dried (yield: 5.23%). The remaining aqueous fraction (AF) was air-dried (yield: 77%). The extract and fractions were stored at 4^º^C until being used.

*Animals*

Male Wistar rats (190 ± 10 g) were purchased from Animal Facility of AJUMS, housed at 20-24^°^C with 12 h/12 h light/dark cycle and free access to food and water. All animals used in this study were treated in accordance with principals and guidelines on Ethics Committee of AJUMS. The rats were deprived of food (not water) for 24 h before the experiment.

*Ileum preparation*

On the day of experiment, rats were sacrificed by a sharp blow on the head and one or two pieces (1.5-2 cm) of ileum were dissected out from 2 cm above the ileocaecal junction and mounted between two stainless steel hooks in tissue bath (10 mL) containing Tyrode solution (37^°^C and pH of 7.4). The lower hook was fixed at the bottom of the tissue bath and the upper one was connected to an isotonic transducer (Harvard Transducer, UK). Each tissue was placed under 1 g resting tension and equilibrated for 60 min prior to the execution of experimental protocols. During this period, the tissue was washed with Tyrode solution every 15 min and the tension was readjusted to 1 g. Ileum contractions were displayed and recorded on Universal Harvard Oscillograph, (UK). Tyrode solution composition consisted of (in mM) NaCl (136.9), KCl (2.6), CaCl_2 _(1.8), NaHCO_3 _(11.9), NaH_2_PO_4_ (0.42), MgCl_2_ (1.05) and glucose (5.55) which continuously was bubbled with air. The ileum contractions were induced by KCl (60 mM) or carbachol (CCh, 10 μM) and once the plateau was achieved, the crude extracts or fractions were added cumulatively (0.125-4 mg/mL of CE and 0.05-0.8 mg/mL of the fractions) to the tissue bath. The mechanism of CF spasmolytic activity was studied by separate tissue incubations either with propranolol (1 µM, 30 min), naloxone (1 µM, 30 min) or N^G ^-nitro - L - arginine methyl ester (L-NAME, 100 µM, 20 min) as non-selective *β*-adrenoceptors, opioid receptors antagonists and nitric oxide synthase inhibitor, respectively. The calcium influx involvement was further evaluated by depolarizing the ileum in Ca^2+^-free Tyrode solution with high K^+^ (60 mM) and then applying CaCl_2_ (0.2-0.6 mM) cumulatively to induce contraction. Same protocol was repeated after incubating the ileum with CF (0.01-0.04 mg/mL) for 5 min. To solve the fractions, DMSO (50 µL) was used and then Tyrode solution was added to make the final solution.

*Chemicals*

CCh, propranolol and L-NAME were purchased from Sigma (Sigma, USA), naloxone was purchased from Tolidaru Company (Tolidaru, Iran) and DMSO was purchased from Merck (Merck, Germany).

*Data analysis*

Plateau of ileum KCl- or CCh-induced contraction was assumed as 100% and the relaxations evoked by extract or fractions, were calculated (as %) and the results were expressed as mean ± SEM for *n* number of animals. Multiple means were compared by one-way and two-way analysis of variance (ANOVA) followed by Dunnett>s test. A p- value of less than 0.05 was considered a significant difference. Statistical analysis was performed using SPSS (version 16.0).

## Results

*Antispasmodic activity of the CE*

**Figure 1:**
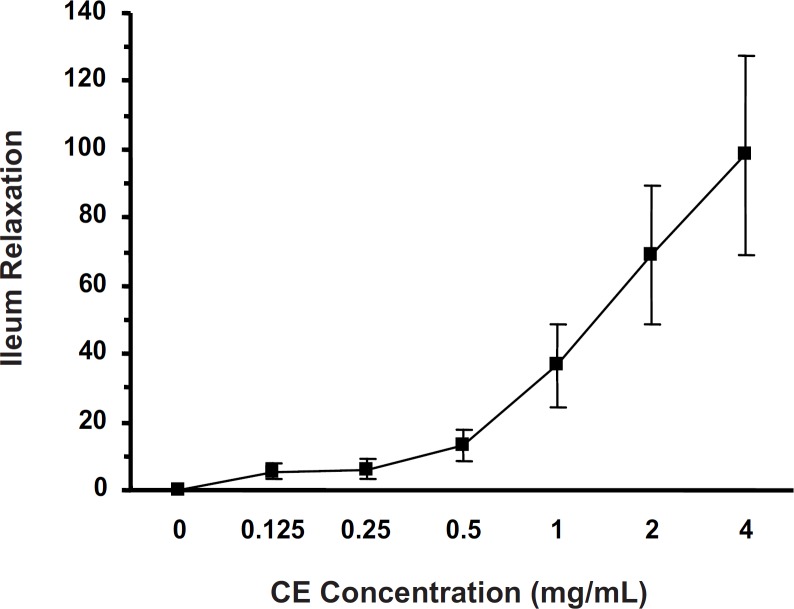
Ileum relaxation (%) induced by crude extract (CE) of *S. lavandulifolia *on KCl (60 mM)-evoked ileum contraction with IC_50_ =0.719 ±0.044 mg/ml (one-way ANOVA, p<0.001).

Cumulative concentrations of CE (0.125-4 mg/mL) attenuated (p < 0.001) the KCl-induced ileum contraction (IC_50 _= 0.719 ± 0.044 mg/mL) in a concentration-dependent manner (Figure 1).

*Antispasmodic activity of fractions*

**Figure 2:**
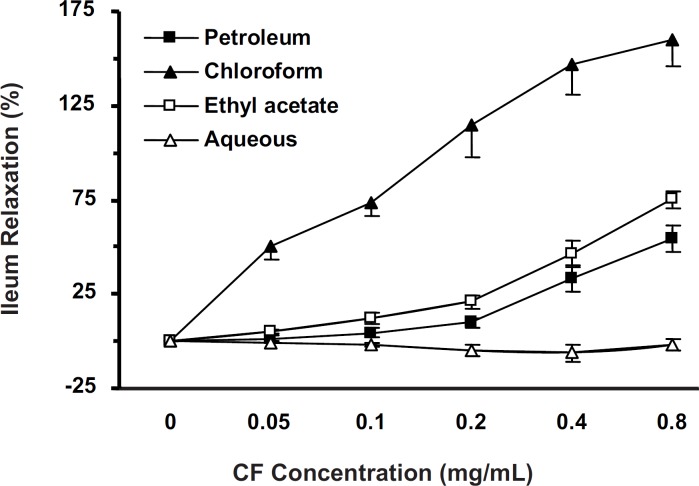
Ileum relaxation (%) induced by *S. lavandulifolia *fractions on KCl-induced contractions. Chloroformic fraction (n = 8) was more potent than PF and EF (two-way ANOVA, p < 0.001). The spasmolytic effects of PF (n = 7) and EF (n = 10) were not different and AF (n = 7) was ineffective

KCl-induced ileum contractions were attenuated with cumulative concentrations (0.05-0.8 mg/mL) of all fractions. PF, CF and EF (but not AF), elucidated antispasmodic activity in a concentration-dependent manner (p < 0.001). The IC_50_ of EF (n = 10), PF (n = 7) and CF (n = 8) were 0.758 ± 0.099, 0.393 ± 0.154 and 0.126 ± 0.018 mg/mL, respectively. PF and EF activities were not significantly different (Figure 2). CF (with lowest IC_50_) was chosen for further studies. 

*Effects of L-NAME, propranolol and naloxone on CF antispasmodic activity*

**Figure 3:**
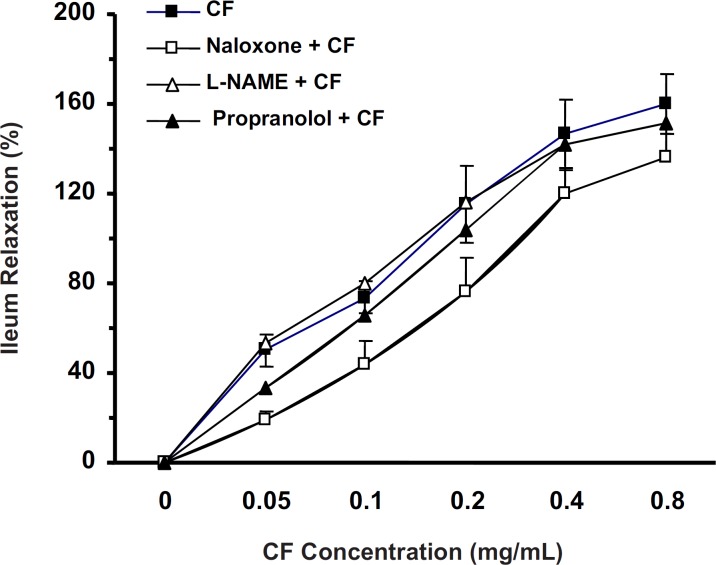
Ileum incubation with naloxone (30 min, 1 µM) attenuated the CF of *S. lavandulifolia *spasmolytic activity (%) in precontracted ileum by 60 mM of KCl (Two-way ANONA, p < 0.01). But CF spasmolytic effect was unaffected by tissue incubation with L-NAME (20 min, 100 µM) or Propranolol (30 min,1 µM).

In the ileum precontracted by KCl, the CF (0.05-0.8 mg/mL) spasmolytic effect was unaffected by tissue incubation with L-NAME (20 min, 100 µM, n = 7) or propranolol (30 min, 1 µM, n = 7). However, naloxone (30 min, 1 µM, n = 9) reduced the spasmolytic activity of chloroformic fraction (two-way ANOVA, p < 0.01) (Figure 3).

*Effect of CF on CaCl*_2_ *-induced ileum contraction*

**Figure 4:**
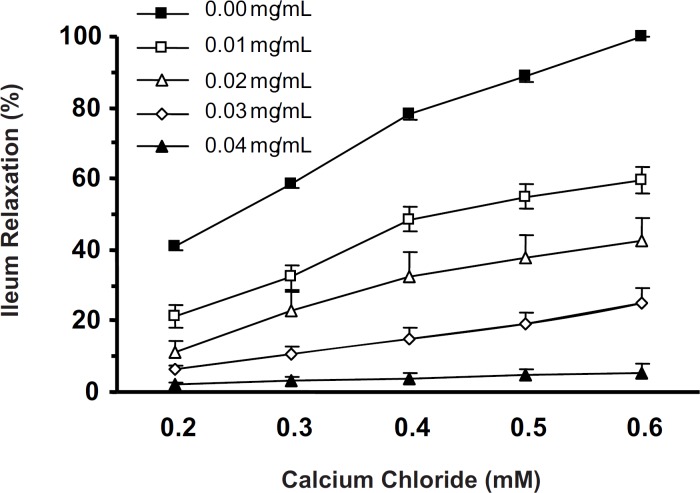
Spasmogenic effect of CaCl2 in Ca^2+^-free, high K^+^ (60 mM) Tyrode solution was attenuated by Ileum incubation (5 min) with CF of *S. lavandulifolia*. The effect of CF at 0.01 mg/ml was significant (two-way ANOVA, p < 0.001).

In Ca^2+^-free with high K^+^ (60 mM) Tyrode solution, cumulative concentrations of CaCl_2_ (0.2-0.6 mM) induced ileum contractions in a concentration-dependent manner (one-way ANOVA, p < 0.001). Five min ileum incubation with CF (0.01-0.04 mg/mL) attenuated the CaCl_2_-induced contractions. The CaCl_2_-induced ileum contractions in the absence and in the presence of lowest concentration of CF (0.01 mg/mL) were significantly different (two-way ANOVA, p < 0.001) (Figure 4).

*Effect of CF on CCh-induced ileum contraction*

**Figure 5:**
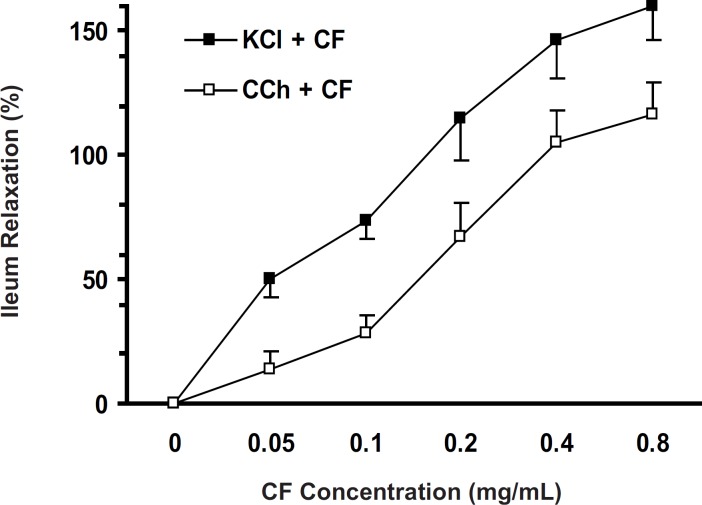
Ileum relaxation (%) induced by CF of *S. lavandulifolia *on KCl-evoked or CCh-evoked ileum contraction. Spasmolytic effect of CF on KCl-induced contraction was more potent than on CCh-induced contraction with IC_50 _= 0.126 ± 0.018 and IC_50 _= 0.184±0.014 mg/ml , respectively (two-way ANOVA, p < 0.05).

The chloroformic fraction (0.05-0.8 mg/mL) attenuated the CCh (10 µM)-induced ileum contraction in a concentration-dependent fashion (one-way ANOVA, p < 0.001) and IC_50_ was 0.184 ± 0.014 mg/mL. The CF antispasmodicactivities on contractions induced by KCl and CCh have been compared in Figure 5.

*Effect of DMSO on KCl and CCh-induced ileum contractions*

Since DMSO was used to solve *S. lavandulifolia* fractions, the possible role of DMSO in the CF antispasmodic activity was studied by applying this solvent to the ileum contractions induced by KCl (60 mM) or CCh (10 µM). Results showed that ileum contractions were unaffected by DMSO (0.005-0.08 w/w).

## Discussion

The most distinctive finding of the present study was the antispasmodic effect of *S. lavandulifolia *on rat ileum. The observed spasmolytic effect was not reversible completely since the ileum responsiveness to spasmogens was not reestablished after refreshing the tissue bath solution. The comparison of spasmolytic effect of crude extract and fractions indicated that chloroformic fraction (CF) with lowest IC_50_ was the most potent fraction. 

The contraction in the smooth muscle is dependent on presence of sufficient cytosolic Ca^2+ ^which can be provided by two main sources: 1) by extracellular fluid mainly via voltage-dependent calcium channels; 2) by releasing Ca^2+^ from intracellular calcium pools such as sarcoplasmic reticulum (7). In the KCl-induced smooth muscle contraction, the voltage-dependent calcium channels (VDCCs) are involved (8) and the existence of L-type VDCCs in rat ileum has been reported (9). On the other hand, CCh (muscarinic receptor agonist) induces ileum contraction via M_2_ and M_3_ receptors (10). Binding CCh to these receptors increases intracellular calcium concentration (11) by both Ca^2+^ influx through L-type Ca^2+^ channels and releasing calcium from cytoplasmic calcium stores (8). The ileum contractions induced by KCl and CCh were not significantly different. CF at 0.2 mg/mL, however, abolished (115%) the KCl-induced contraction but relatively inhibited (66%) the CCh-induced contraction. Thus, the KCl-induced contraction was more susceptible to CF than the one elicited by CCh, which suggested that events at the cholinergic receptors level were not important.

It has been suggested that the substance that inhibits the KCl-induced smooth muscle contraction is considered as a Ca^2+^ influx blocker (12). The result of CF spasmolytic effect on CaCl_2_-induced contractions supports this suggestion, since high K^+^ in the Ca^2+^-free Tyrode solution depolarizes the ileum (13) and the contraction may occur only after applying CaCl_2_ (11). Furthermore, KCl and CCh increase intracellularCa^2+^ by activating the calcium channels which suggests that CF of *S. lavandulifolia *has inhibited Ca^2+^ mobilization.

Activation of *β*-adrenoceptor activation evokes the ileum relaxation (14). The ineffectiveness of propranolol in attenuating the CF spasmolytic effect, however, indicates that *β*-adrenoceptors were not involved in CF activity. 

Nitric oxide as a main inhibitory neurotransmitter in the gut (15) modulates the contractility of small intestine through cGMP (16) but, the ineffectiveness of L-NAME (nitric oxide synthase inhibitor) to attenuate the CF spasmolytic effect indicates that CF activity was not mediated through nitric oxide. Opioid receptors activation relaxes the ileum smooth muscle (17) and in our study, naloxone (non-selective opioid receptors antagonist) partially but significantly reduced the CF effect which suggests that CF activity has been mediated, at least partially, via these receptors. Furthermore, the KCl- and CCh-induced ileum contractions were unaffected by DMSO indicating that the observed CF spasmolytic activity was not due to DMSO effect.

The main constituents of *S. lavandulifolia*are alpha-pinene, myrcene, *β*-phellandrene and *β*-caryophyllene (4) whichsome of them have spasmolytic effect (18, 19). The existence of apigenin and luteolin in *S. lavandulifolia* (20) and the antispasmodic effects of these two flavonoids have been demonstrated (21-23), therefore, it seems that the observed spasmolytic effect of *S. lavandulifolia* was due to its flavonoid constituents. Relatively low IC_50_ of the chloroformic fraction of *S. lavandulifolia*suggests possible beneficial effects of this herb on which, however, further research is needed to be confirmed. 

In conclusion, *S. lavandulifolia* Vahl. chloroformic fraction of aerial parts, particularly, induced spasmolytic effect in the isolated rat ileum mainly by disturbing calcium influx and partly via opioid receptors activation. The obtained results support the traditional usage of this herb to treat diarrhea.
